# The Worth of Inflammation
*"The Lessons of Acute Disease." H. Cameron Gillies, M.B, London. (David Nutt, One shilling net.)


**Published:** 1892-11-26

**Authors:** 


### THE WORTH OF INFLAMMATION.*

When you are told that any one is suffering from inflammation of lungs, or brain, or any other organ, or if he has met with an injury and inflammation has set in, it is always implied in the statement not merely that the patient is suffering from acute disease, but that the inflammatory process is an important part of the complaint, if not the whole of it. The idea is not a monopoly of the ignorant; it is shared and spread by many of the medical profession, who do all in their power by drugs and diet to check the inflammation. It is certain that inflammation has an ugly and alarming appearance, but all ugly things are not necessarily bad or dangerous; some, indeed, are benignant safety-valves or drainage tubes by which much that is truly evil gets away. A young mother is distressed at the eczema that appears on her teething baby, but if, before sending for the doctor, she consults some old woman who has brought up a dozen children, she will certainly be told, "Don't drive it in, or it will be the worse for the child." Then will follow tales of convulsions and hydrocephalus which followed the "driving in" in the narrator's experience, which need not be detailed, and perhaps have not a very close connection with the advice given; but, nevertheless, the advice is sound—"Don't drive it in; don't, for the sake of getting rid of the ugly 'rash,' check the inflammatory process which is carrying a poison out of the system." In measles, chicken-pox, and small-pox no one would think of suppressing the eruption; it is allowed to do its beneficient work; but it has not yet occurred to most people to trace any connection between these and other forms of inflammation. They are not indeed alike in all points, but all inflammation is primarily an attempt by Nature to repair injury, and to eliminate waste and poisonous substances. If doctors snub Nature and try to check her efforts she is apt to sulk. "Driven in upon herself she became morbid." This sentence might apply to the gloomy and unappreciated heroine of any novel of the day; it is an accurate statement of the conduct of Nature when her well-meant efforts are misunderstood and suppressed. She becomes morbid; she gives death instead of life.

Inflammation is not a disease, except in the literal sense of discomfort. It is rather an accompaniment of disease, like the doctor, the nurse, and the medicine-bottle; and is, in itself, no more formidable than these. It comes as they do, in the train of a condition of health, or rather lack of health, which should never have existed, but like them it comes to cure and save. In some diseases we realise this, though unconsciously. Does any doctor, when called in to a case of rheumatic fever, devote his chief attention to reducing the swollen joints? Knowing that the fever is caused by an excess of lactic acid in the blood, he wraps his patient up in flannel and cotton-wool to prevent any risk of chill; for the chief point is to keep the pores open, and let the acid perspiration, charged with the poisonous acid, come out of the body as quickly as possible. After that—well, after that there will not be much inflammation to deal with. Nature's efforts have been supplemented by man; and he and she may rest from their work together. To cure the cause which rendered inflammation necessary—this is the doctor's work, and not the cure of inflammation itself. And indeed this should not be the doctor's work, but the possible patient's who should keep himself in such health that there shall be no need for inflammation to "set in," and try to cure his physiological sins.

The inflammation that comes from injury might be regarded as of a different kind, but fundamentally it is the same. Inflammation is a supply of tissue building material which comes to any part that needs it, either to supply loss occasioned by injury, or to restore to a proper condition tissue that has been weakened by disease. In each case there is loss, but the loss of blood from an open wound is more easily seen than in the anæmic condition which culminates in pneumonia or typhoid fever, and therefore people fail to guess at any similarity between them. But in each case the so-called inflammation is simply an active process of repair. No wound heals without inflammation. This may be doubted, for sometimes, it is true, the inflammation is so slight that it is not perceived. A straight cut with a sharp clean knife will heal with no perceptible swelling if the parts are at once put together and allowed to rest. But even in healing "by the first intention," as it is called, some material is required to unite the severed parts, as cement is required to mend broken china, with this difference, however, that the living organism itself secretes and brings into use the cement it needs. When the cut is wider, with ragged, gaping mouth, a larger supply of cement is needed—"inflammation sets in." Perhaps this statement may be contradicted. We may be told that it is not inflammation but granulation that has appeared, and granulation is an admittedly beneficial process. But it passes the wit of man to say wherein lies the difference between the two. Sir James Paget, in describing granulation, is forced to say that "It is beneficial in its end or purpose but morbid in its method, being comparable with the process of inflammation more than with any of those that are natural to the body." He holds that inflammation is as bad in its action as granulation is good, though the two are so much alike that the one can only be described by comparison with the other. Yet it never seems to have occurred to him that they may be practically identical, that they may be at most only different degrees of the same reparative process.

As with a wound on the surface of the body, so is it with a lesion within. Where there is inflammation (or internal granulation, if that term be preferred), the work of cure is going on. New tissue material is being brought to repair or supplant that which is worn out; waste matter, poisonous in its uselessness, is being pushed out of the way and disposed of by the natural organs of secretion, when that is possible; if not, by abscesses and other special organs made for the occasion. It is no wonder that inflammation is so maligned when it comes with such an ugly companion as an abscess; yet this, too, means no ill, but rather good, carrying to the surface of the body, where it can be easily got rid of, the morbid stuff that would otherwise have poisoned the system. In fact, we may doubt if most of the things which we now call diseases are not really Nature's methods of cure. The real diseases were antecedent to these—over-work, self-indulgence, exposure, neglect. Let us direct our efforts to preventing these, or if we cannot do this, let us at least in our cures follow Nature's guidance, instead of trying to obstruct her path.

* "The Lessons of Acute Disease." H. Cameron Gillies, M.B. London. (David Nutt. One shilling net.)

